# Extracts from a Discourse on the Disease Called Tic Douloureux, Read before the District Medical Society, at Boston, in April, 1812

**Published:** 1814-03

**Authors:** James Jackson


*For the Medical and Physical Journal.*

*Extracts from a Discourse on the Disease called Tic Douloureux, read before the District Medical Society, at Boston, in April,* 1812. By James Jackson, M.D.

*A brief Account of the Notices which have been taken of this Disease.*

THERE is not any reason to doubt that this painful disease has existed in all ages. It is not, however, described by any of the Greek writers on medicine. Among the Ara-

 bians bians it is said to have been observed. We are told that Rhazis, one of the most celebrated physicians among the Arabians, has transmitted to us a description of this disease, for which he gives credit to Simion Seth, physician to one of the emperors of the east, and who lived in the eleventh century. Subsequent to this period, Ebr Sina, of the same nation, described the disease more fully, and with more precision.*

Whatever may have been the merit of the description of these Arabians, it is certain that the disease was not distinctly recognized by the faculty in general until after the middle of the last century. It was in France that it received the earliest attention. A surgeon of Versailles, named Andrè, published in 1756 a collection of chirurgical observations, among which there were some respecting the tic douloureux.† It is probable that we owe to Andrè this name; at least it is to him we trace its use. About twenty years after this time (1773) Dr. John Fothergill communicated to the London Medical Society his remarks on the disease, to which he gave the appellation of "a painful affection of the face." Dr. Fothergill does not appear to have been aware that the disease had ever been noticed by any one before himself; and his description, which is very accurate, is drawn from his own observations on a small number of patients. The description given by Andrè I have never seen and cannot procure, and therefore I am not authorised to give any very positive opinion respecting it; but with the medical world in general, and even some of the French writers, I am disposed to give to Fothergill the credit of having first described this disease. For, although it had before been described so that we can now recognize it in the writings of his predecessors, yet it was never before so described, as that physicians in general were made aware of its existence as a separate disease, and of its peculiar characteristics. From the time of Fothergill every one has been prepared and enabled to discover this painful affection wherever it has been seen.

Since Fothergill's publication (in the Medical Observations and Inquiries) many writers have noticed the subject. Pujol, a French physician, published an essay upon it in 1787, which is said to have been very learned, and to have evinced great research among ancient and modern writings for traces of

---

* Diagnost, med. and Recueil periodique de la Soc. de med. de Paris. Tom. 4.

† Traité du diagnostic medical par Dressig, traduit de l'allemand par L. J. Renauldin, a Paris. 1804.

this this disease under some other name. These researches were not very profitable; but a knowledge that they have once been made with accuracy by a man of learning, is useful, as it may prevent a loss of time in a repetition of them by others. Previous to Pujol's essay the subject had been considered in a memoir by Thouret and Andry, presented to and published by the Royal Society of Medicine of Paris (1779), and since Pujol, by other writers in France and Germany. In England also the disease has been noticed in periodical publications, and in distinct works; particularly by Dr. Haighton (Medical Records and Researches), and by Dr. S. Fothergill, nephew to the Fothergill before mentioned.

In this country the disease does not appear to have arrested the public attention for any great length of time. Of late there has appeared an interesting and distressing case, which occurred in a very respectable physician of New-York.* It is not a little remarkable that the disease seems to have been quite unknown in that city, previous to the case here referred to.

Besides the names for this disease which have already been mentioned, (tic douloureux, and painful affection of the face,) others have been proposed; of which, one given by Professor Chaussier appears to be the best. This is *neuralgia of the face.*

*Description of the Disease.*—This disease is characterised by an exceedingly violent pain, which affects some part of the face for a short space of time, and recurs at uncertain intervals. It is very sudden in its attack, and is sometimes† perceived distinctly to pursue the course of the ramifications of some nerve.

This neuralgia is common to persons of both sexes; but has very rarely been noticed in any previous to the adult age, and is not common in persons under forty years of age. It has, however, been noticed in those between twenty and thirty years of age. It does not appear, on comparing the observations of different writers, that persons of any particular temperament or constitution, or that those who have pursued any particular employment or mode of life, are especially subject to this disease. I am disposed to think that the pain is usually more violent when it occurs in persons much advanced in life, than when in young persons.

The seat of the disease is for the most part in the face, but

---

* See Medical Museum, New Series, Vol. I. No. II.

† This circumstance is not so constantly to be observed as the description of some good writers would lead us to think.

in some instances it extends over the head. Very often it is over the antrum highmorianum, and spreads to the ala nasi and upper lip. It spreads also to the gums, and even to the roof of the mouth.

In other cases it passes from the ear over the cheek, and this is very often its seat. Less frequently it is seated in the lower jaw; and occasionally it branches out from above the eye over the forehead. It has also been observed to affect the mastoid process and the occiput. Originating in some one of the places which have been described, it very often extends to all the others. It sometimes darts from the proper seat of disease to the surrounding parts with great rapidity, "shooting like radii from a centre."

It very rarely happens that both sides of the face are affected. This has, however, been said to occur in one or two cases. The disease has been known to affect each side alternately; and it has been thought to alternate with convulsive affections of other parts of the body.

Some writers state that the paroxysms occur only in the day. It is however certain that they do occur in the night,* and even most severely in some instances.

The duration of the disease is uncertain. The paroxysms are mostly sudden in their access; but sometimes there are precursors. The pain is variously described. Very often it comes on by strokes or touches, like a spasmodic affection. These continue only a few seconds, or never perhaps more than a minute; but the frequency of repetition is exceedingly various. The repetition is sometimes so frequent, that the pain seems almost constant. It is more frequent during certain periods; and these periods are from a few days to several weeks in duration. The intervals between these periods are also various; continuing a week, a month, a year, or even longer. There are patients who seldom obtain any of these respites from their distress, having some pain constantly; while others are perfectly at ease during these intervals. The suffering is excruciating in degree; being such "as neither words can describe, nor the imagination easily conceive."

The pain is many times produced by evident exciting causes. At others it arises without any warning, and, as it would seem, spontaneously. There is this peculiarity respecting it: it will frequently be produced upon very slight irritations. A light touch, as in knocking off a fly, or in moving the bed-clothes across the face, will in some patients occasion very excruciating pain; while the same persons can

* Diagnost. Medical.

bear bear to press, or strike upon the part affected with great violence, without any unpleasant effect. The necessary motions in eating and speaking very often occasion a return of the paroxysms; as indeed will any spontaneous motion of the parts affected. Accordingly in severe cases the patient will endeavor to avoid any, the slightest motion of those parts; and in consequence of this restraint the countenance acquires a peculiar expression. The muscles of one side of the face only are put into action; but the effect is very different from what happens in hemiplegia, seeing that the power is not lost, and the restraint is voluntary and even forced. The patient will take in his food only on the sound side of the mouth. The food must be such as not to require mastication, for this process cannot be performed without risk. Even in swallowing liquids, or food of the softest texture, the pain is, in the worst cases, so excruciating, that the patient restrains himself to the least possible quantity; not thinking of gratifying the appetite, but endeavoring only to appease the most imperious calls of nature. In speaking, also, the patient must, during the periods of severe pain, be equally cautious and abstemious, uttering only monosyllables, or the shortest sentences; and in doing so much he restrains one half of the mouth from motion, so far as it is possible.

This terrible disease remits, and occasionally subsides for ever, in a very sudden manner. It is often relieved for a time by engaging the attention of the mind. One gentleman, it is said, found relief in the amusement of cards.*

It is commonly said that this disease is not attended with any swelling, or change of structure. This in general is true; but in some cases a general tumefaction of the gums takes place during the paroxysms of pain. See cases of Mrs. W. and Mrs. C.

Diagnosis.—This neuralgia of the face is similar in some respects to four other diseases. It is therefore necessary to consider how it may be distinguished from them. These four diseases are trismus, odontalgia, rheumatism affecting the muscles of the face, and hemicrania.

*Symptoms which distinguish the Neuralgia of the Face from Trismus.*—In the first of these diseases we find pain the most strongly marked symptom; although sometimes convulsive actions are observed about the face, they are proportionally slight. In trismus the spasmodic contraction of the muscles of the lower jaw constitutes the essential part of the disease; and, although this is attended with some pain, it is very inferior to that of neuralgia.

* See Dr. Fothergill's Treatise on Tic Douloureux.

In the first of these diseases the jaw is sometimes kept nearly closed, so that the patient does not admit any, except very small portions of solid matter, to pass between his teeth; nor does he perform the action of mastication. But it is evident that all this arises from a dread of producing pain by a more free motion of the parts, and not, as in trismus, from an involuntary contraction.

*Symptoms which distinguish Neuralgia of the Face from Odontalgia.*—One mode which has been mentioned of distinguishing between these diseases is from a consideration of the age of the patient. It has been thought that the disease rarely, if ever, appears until after the fortieth, and not often until after the fiftieth year; at which period of life the teeth are seldom the seat of pain, even if there remain any in their sockets. But I apprehend that too much reliance must not be placed on this mode of discrimination. Forstmann, a German writer, quoted with great respect by Heurteloup in the collection of the Society of Medicine of Paris,* says that he has seen the disease in young girls of the age of twenty, and even of nine years. Heurteloup also gives a long and minute account of a case in a Corsican, who was under thirty years of age. Darwin also, in his Zoonomia, gives a strongly marked and very severe case, in which the patient was between twenty and thirty years of age.

From the age alone, then, we are not authorised to decide the nature of the painful affection. But another question has arisen in my mind on this subject, and this is, whether affections of the teeth do not sometimes produce the neuralgia of the face. The pain peculiar to the latter disease certainly bears a resemblance to the pain which sometimes occurs in affections of the teeth, and which is described as jumping, twinging, &c. although the latter is seldom, if ever, comparable in severity to the neuralgia. But it is not on this resemblance alone that I have been induced to raise a question on this point. The question first arose in my mind in consequence of a case which occurred to me two years since, and which I will take the liberty to relate.

*Case of Mrs. S.*—I was called to this lady in April, 1810, and then learnt from her the following history. In 1806, she had one of the molares removed from the upper jaw on the left side. It was the fourth, and at that time the last of those

* Recueil period. de le Soc. de Med. de Paris, Tom. 4, p. 205. "Cette dissertation de Forstmann est savante, pleine d'erudition et merite de tenir place parmi les bons écrits publiés sur le tic douloureux de la face. On y trouve les examples de jeunes filles de neuf et de vingt ans attaquées du tic douloureux."

teeth teeth. A portion of the socket was removed, adhering to the tooth. The operation occasioned great pain; and this continued very severe for some time afterwards. At the end of three or four weeks the parts were inflamed; and a physician who saw her thought that he could perceive a loose portion of bone. The inflammation subsided, however, without the discharge of any bone.

About seven months after the extraction of the tooth, she first experienced in the socket a pain peculiar in kind, and excruciating in degree. This came on very suddenly in the night. It continued to distress her for some days at that time, and recovered at several subsequent periods, continuing from half an hour to many days. In this time she had consulted different gentlemen of the faculty, and two of them, on examining the socket, had told her that a tooth was coming, and she had in consequence several times had the gum divided very freely.

When I saw her I found an enlargement of the socket, and this was very hard. The appearance was such, that I should at once have adopted the opinion that a tooth was contained within the socket, had I not been told that the parts had then continued in the same state for more than three years. The pain at this time was excruciating. It had commenced some days before, and had been growing more constant. The description of the pain was this. Its access was sudden and violent, and it rarely continued so long as five minutes, sometimes not one, without a sensible intermission. This intermission was of various duration, from one or two minutes, to hours, days, &c. But the pain was not strictly constant during the short period described. It consisted of successive strokes or shocks, very quickly repeated. These were darting, and more severe than words can describe, and they spread from the socket to the cheek and ear, and occasionally over the side of the head. The miserable patient was unable to lie down when suffering the pain, and as this continued in the night as much as in the day, she often spent a great part of the night out of bed. At one time, she passed nearly a week without having more than one hour's sleep in a night on an average.

I divided the gum without affording her any relief, although previously she had got some ease after this operation. Internally, I administered first the aq. ammoniæ without relief; I then directed the inspissated juice of *hemlock* (conium maculatum), and after increasing it to a very large dose, such as affected her head, she was relieved. The pain occasionally returned for short periods, during three or four months months afterwards. The same remedy would always give relief.

Six or eight months after she had felt any of the pain, she found that a tooth had protruded from the part where the tumor had been, with so little uneasiness that she had not discovered it, until fully grown. Since that time she has continued perfectly free from her distressing pain.

In this instance the pain had the most important characteristics of the neuralgia. It was uncertain in the time of its attack, sudden, coming by strokes, or darting, and excruciating in degree. It was also mostly, though not wholly, confined to the portio dura of the seventh pair of nerves. At the same time it is impossible not to attribute the pain to the tooth, which was for so long a time retained under the gum.

To return from this digression, let us proceed to consider the signs by which the tooth-ache may commonly be distinguished from the tic douloureux.

One obvious and important distinction is to be derived from the seat of the pain. In the odontalgia the pain is in the teeth, or sockets, extending, however, by continued sympathy to the neighbouring parts in severe cases. In the neuralgia it follows the course of some one, or more considerable nerves.

In odontalgia there is commonly to be discovered some obvious cause, as a carious tooth, or a tooth piercing the gum, or pregnancy; and where there is a carious tooth, the removal of it gives relief. But in neuralgia the pain is rather increased than diminished by the removal of the teeth; and the disease occurs in subjects who have lost all their teeth.

It is said that the odontalgia is never accompanied by convulsive motions in the muscles of the face; while these frequently manifest themselves in neuralgia.*

*Symptoms which distinguish Neuralgia and Rheumatism of the Face.*—The rheumatic affection is said to have regular periods of access, and those mostly at night; while the neuralgia occurs at irregular periods, but those in the day time. In most cases, perhaps, it is worse in the day time than in the night, possibly owing to the inevitable operation of many exciting causes during the day. However, the neuralgia does occur with great severity in the night, although some authors have thought otherwise.

The pain of rheumatism is more constant and much less severe than that of neuralgia. When rheumatism is most

* Traité du diagnostic medical.

severe, severe, as when it is acute, it is usually accompanied by swelling of the parts affected, which does not occur in any considerable degree in the other disease.

Finally, the rheumatic pain does not regard the distribution of the nerves; while the neuralgia follows them so exactly, in some cases, that their distribution may almost be learnt by attending to the course of the pain.

*Symptoms which distinguish the Neuralgia from Hemicrania.*—There is certainly a great resemblance between these two diseases; so great, that Dr. Curry, a physician of Guy's Hospital, does not hesitate to declare, that they are one and the same disease. "I am now fully convinced," says he, in a letter to Dr. S. Fothergill,* "that the disease commonly known under the name of hemicrania, is no other than the tic douloureux affecting the nerves which supply the temporal muscle and scalp." It would require too much time to examine the evidence offered by Dr. Curry in support of his opinion. It certainly has some weight, but it is not conclusive.

Hemicrania is most commonly intermittent, occurring in paroxysms, as regular as those of an ague. This is not a very rare disease with us in spring and autumn, and sometimes it occurs in winter. It is frequently a concomitant of a catarrhal affection. The pain is generally in one temple or about one eye, occasionally spreading over the side of the head, but seldom or never extending to the cheek. It is usually quite confined to one side of the head. It does not occur at any one stated hour in all cases; but in most it comes on about ten or eleven o'clock A.M. and continues from four to eight hours. From the time of the access it gradually increases until near its termination; for after rising to its greatest height, it subsides pretty rapidly. It yields to cinchona; but more certainly to arsenic, employed as directed by Fowler.

This disease is very painful, but the pain is not of the lancinating kind which occurs in the neuralgia. It may certainly be easily distinguished from this last.

(To be continued.)

---

*For the Medical and Physical Journal.*

*Case of severe Luxation of the Jaw reduced; by* Mr. A. Kington.

ON Christmas-day last, I was requested to visit Thomas Vaughn, a strong muscular man, who was frequently the subject of a dislocated jaw. Upon my arrival, I found

† Dr. S. Fothergill on Tic Douloureux, page 73.

